# Cognitive Functioning and Walking Speed in Older Adults as Predictors of Limitations in Self-Reported Instrumental Activity of Daily Living: Prospective Findings from the Obu Study of Health Promotion for the Elderly

**DOI:** 10.3390/ijerph120303002

**Published:** 2015-03-11

**Authors:** Hyuma Makizako, Hiroyuki Shimada, Takehiko Doi, Kota Tsutsumimoto, Sangyoon Lee, Ryo Hotta, Sho Nakakubo, Kazuhiro Harada, Sungchul Lee, Seongryu Bae, Kenji Harada, Takao Suzuki

**Affiliations:** 1 Department of Functioning Activation, Center for Gerontology and Social Science, National Center for Geriatrics and Gerontology, 7-430 Morioka-cho, Obu, Aichi 474-8511, Japan; E-Mails: shimada@ncgg.go.jp (H.S.); take-d@ncgg.go.jp (T.D.); k-tsutsu@ncgg.go.jp (K.T.); sylee@ncgg.go.jp (Sa.L.); ryo-h@ncgg.go.jp (R.H.); sho-n@ncgg.go.jp (S.N.); haradak@ncgg.go.jp (Ka.H.); leesuys@ncgg.go.jp (Su.L.); bae-sr@ncgg.go.jp (S.B.); harada-k@ncgg.go.jp (Ke.H.); 2 Research Institute, National Center for Geriatrics and Gerontology, 7-430 Morioka-cho, Obu, Aichi 474-8511, Japan; E-Mail: suzutaka@ncgg.go.jp

**Keywords:** instrumental activities of daily living, memory, processing speed, walking speed

## Abstract

Our aim was to determine whether baseline measures of cognitive functioning, walking speed, and depressive status are independent predictors of limitations in instrumental activities of daily living (IADL) in older adults. The cross-sectional study involved 1329 community-dwelling adults, aged 75 years or older. At baseline, the Mini-Mental State Examination (MMSE), Symbol Digit Substitution Test (SDST), Geriatric Depressive Scale (GDS), and a word list memory task were completed, and self-reported IADLs and walking speed were recorded. The longitudinal study involved 948 participants without baseline IADL limitation, which was assessed at baseline and 15-month follow up, using the three Kihon Checklist subitems. In cross-sectional analyses, participants with IADL limitation demonstrated greater GDS scores, slower walking speeds, and lower MMSE, word list memory task, and SDST (only for women) scores relative to those without IADL limitation. In the longitudinal analyses, baseline walking speed (men: OR 0.98; women: OR 0.97, *p* < 0.05) and word list memory task scores (men: OR 0.84; women: OR 0.83, *p* < 0.05) in both sexes and SDST scores in women (OR 0.96, *p* = 0.04) were independent predictors of subsequent IADL limitation. Walking speed, memory, and processing speed may be independent predictors of IADL limitation in older adults.

## 1. Introduction

The loss of the ability to conduct daily activities leads to a rise in morbidity, caregiver burden, and mortality [1,2]. Generally, everyday functioning ability in older adults consists of separate assessments of basic activities of daily living (BADLs) and instrumental activities of daily living (IADLs). BADLs are self-maintenance skills, such as bathing, dressing, and toileting, whereas IADLs are also routine activities, but they are more goal oriented and related to more complex and higher functional abilities, such as preparing a meal, handling finances, shopping, and other activities [3,4].

Older people with IADL limitations are frailer, because they have a greater number of associated health problems, such as disorders (e.g., histories of heart disease, stroke, depression, or diabetes), poorer cognitive function, and frequent falls [5]. Conversely, physical and cognitive status could also be predictors for the onset of IADL limitation in older people [6,7].

With respect to associations between cognitive function and IADL difficulty, a few longitudinal studies have focused on specific neuropsychological domains and examined causal relationships between multidimensional cognitive function and future decline in IADLs. For instance, Johnson and colleagues performed a longitudinal study and showed that baseline executive functioning was associated with future worsening of IADL dependence [8]. Other studies have also examined associations between baseline cognitive domains, including memory or executive function, and a decline in IADLs in older people [9,10]. Taken together, baseline cognitive functioning, particularly executive function and memory, appear to be important predictors of the onset of IADL limitation.

In contrast, physical function is strongly associated with the ability to perform IADLs [11]. In particular, aspects of walking ability, such as walking speed, could be strong predictors for the onset of IADL limitation in aged populations [12,13]. In addition, depressive symptoms may be a predictor for an increase in IADL limitation [14]. However, no studies have examined the means by which specific cognitive functions, including concurrent walking ability and depressive status in older adults, predict future IADL limitation.

Therefore, we sought to determine whether baseline measures of cognitive functioning are independent predictors of subsequent IADL limitation, with walking speed and depressive symptoms considered covariates, in community-dwelling older adults aged 75 years and over with no IADL limitation at baseline. We hypothesized that the association between cognitive function and future IADL decline would be independent of age, sex, education, medication, depressive status, and walking speed.

## 2. Methods

### 2.1. Participants

All of the participants in this study were enrolled in the Obu Study of Health Promotion for the Elderly (OSHPE) [15]. A letter of invitation was sent to all older individuals living in Obu, a residential suburb of Nagoya, Japan, to participate in the OSHPE. Excluded were participants requiring support or care certified by the Japanese public long-term care insurance system (care level ≥3/5) and those in similar ageing cohort studies. At baseline, 1392 community-dwelling older adults aged 75 years or older participated in the study. We conducted a baseline assessment for the OSHPE, including a face-to-face interview and measures of physical and cognitive function (August 2011 to February 2012). A follow-up postal survey was conducted approximately 15 months after baseline assessment (November 2012 to May 2013), with an offer of assistance in completing the study. In the cross-sectional study, we excluded participants with a history of Parkinson’s disease, stroke, Alzheimer’s disease, or depression. Participants with missing values for education level (n = 4), depression symptoms (n = 4), walking speed (n = 13), cognitive function (n = 31), and self-reported IADLs (n = 11) at baseline were also excluded. In this prospective study, we included participants who had completed baseline measures of walking speed and cognition and follow-up assessments of self-reported IADLs. Of the participants included in the cross-sectional study (N = 1329), participants who could not complete self-reported IADL assessment in a follow-up postal survey (n = 247) or suffered a stroke, Alzheimer’s disease, depression, or a hip fracture (including those for whom onset of the disease was unknown) subsequent to baseline assessment (n = 134) were excluded. Ultimately, 948 participants without IADL limitation at baseline were included in the current prospective cohort study. Informed consent was obtained from all participants prior to their inclusion in the study, and the ethics committee of the National Center for Gerontology and Geriatrics approved the study protocol.

### 2.2. Measurements

Demographic data including age, sex, and number of medications used on a regular basis were recorded during face-to-face interviews at baseline. The participants completed a standardized questionnaire, which included the self-rated 15-item Geriatric Depression Scale (GDS) [16].

IADL limitation was assessed using the three subitems of the Kihon-Checklist [17], a self-reported comprehensive health checklist that was developed by the Japanese Ministry of Health, Labour and Welfare [18], at baseline and 15-month follow up. The three sub-items are as follows: (1) using the bus or train by myself, (2) buying daily necessities by myself, (3) managing my own deposits and savings at the bank by myself. In the cross-sectional study, a participant response of “no” to one or more items at baseline assessment represented IADL limitation. In the longitudinal study, none of the participants reported IADL limitation in any of the three sub-items at baseline. Participants with no IADL limitation, according to their responses to a 15-month follow-up survey, were considered independent, and those who reported one or more IADL limitations in the 15-month follow-up survey were assigned to the IADL-limitation group.

We measured baseline walking speed in seconds, using a stopwatch. Participants were asked to walk 6.4 m (divided into two 2.0 m zones at each end and a 2.4 m middle zone) at their usual pace. We measured the time required (in seconds) to pass the 2.4 m middle zone over five trials in order to calculate mean gait speed (meter per minute: m/m).

Participants also underwent measures of global cognitive function, verbal memory, executive function, and processing speed using the National Center for Geriatrics and Gerontology Functional Assessment Tool (NCGG-FAT) [19]. Global cognitive function was assessed using the MMSE translated into Japanese [20], which is based on the original version [21]. Verbal memory was assessed using delayed recall in a word list memory task. Before delayed recall was tested, participants were instructed to memorize 10 words that were presented on a tablet PC. Each of the 10 target words was shown for 2 s. A total of 30 words, including 10 target and 20 distracter words, were then presented, and participants were asked to choose the 10 target words immediately. This was repeated for three trials, and participants were instructed to recall (write down) the 10 target words following a delay of approximately 20 min. We calculated the total number of recalled target words. One point was awarded for each word recalled correctly within 60 s, with a maximum score of 10. We used the tablet version of the symbol digit substitution test (SDST) to assess processing speed. In this task, nine pairs of numbers and symbols were presented at the top of the display. A target symbol was presented at the center of the display. Participants then chose a number that corresponded to a target symbol at the bottom of the display as rapidly as possible. The score comprised the sum total of the correct numbers chosen within 90 s. One point was given for each number chosen correctly within the time limit. Higher scores represented better performance. A previous study reported that SDST and the delayed recall word list memory task demonstrated excellent test-retest reliability and validated the test in comparison with scores on widely used conventional neurocognitive tests (*i.e.*, the subtest of the Alzheimer’s Disease Assessment Scale-Cognitive and the Digit Symbol-Coding subtest of the Wechsler Adult Intelligence Scale (Third Edition)).

### 2.3. Statistical Analysis

Data entry and analysis was performed using SPSS version 19.0 (SPSS Inc., Chicago, IL, USA). A *p* value of <0.05 was considered indicative of statistical significance. Means, standard deviations, and proportions were calculated to describe the samples and provide summary information regarding the measures used. Student’s *t* tests were used to compare differences in measures between the independent and IADL-limitation groups at baseline in the cross-sectional study. Chi-square tests were used to compare differences in rates of IADL limitation onset between men and women during the 15-month follow-up period. We also compared baseline measures between the independent and IADL-limitation groups using Student’s *t* tests in the longitudinal study.

Logistic regression analysis was performed to examine whether potential determinants were independently associated with subsequent IADL limitation. In this analysis, subsequent IADL limitation was included as a dependent variable, and age, education level, number of medications used, GDS score, walking speed, MMSE, word list memory task scores, and SDST scores were included in the model as independent variables. Odds ratios (ORs) and 95% confidence intervals (CI) were calculated for the subsequent IADL limitations.

## 3. Results

Results showed that 139 (21.8% of 638) older men and 114 (16.5% of 691) older women reported IADL limitation at the baseline assessment. Table 1 shows characteristics, GDS, walking speed, and cognitive function comparisons between participants with and without IADL limitation at the baseline assessment. Men with IADL limitation exhibited significantly lower education levels, greater GDS scores, and slower walking speeds relative to those observed in men without IADL limitation (*p* < 0.01). Women with IADL limitation were significantly older than men with IADL limitation; they also exhibited significantly lower education levels, greater GDS scores, and slower walking speeds than those observed in the men (*p* < 0.01). With regard to cognitive performance tests, men without IADL limitation exhibited significantly higher scores on the MMSE (*p* = 0.024) and SDST (*p* < 0.01) than those observed in men with IADL limitation. MMSE, word list memory task, and SDST scores in women without IADL limitation were significantly higher than those observed in women with IADL limitation (*p* < 0.01).

**Table 1:** Comparison of characteristics between participants with and without self-reported IADL limitation in the baseline survey (n = 1329).

Variable	All Participants (n = 1329)	Men (n = 638)				Women (n = 691)		
		Independent (n = 499)	IADL Limitation (n = 139)	*p*-Value		Independent (n = 577)	IADL Limitation(n = 114)	*p*-Value
Age in years	79.2 ± 3.9	79.2 ± 3.7	79.2 ± 4.6	0.991		78.9 ± 3.7	80.9 ± 4.2	<0.001
Education in years	10.5 ± 2.6	11.3 ± 3.0	10.2 ± 2.7	<0.001		10.0 ± 2.1	9.4 ± 2.0	0.006
Number of medications used	2.5 ± 2.3	2.3 ± 2.3	2.7 ± 2.7	0.054		2.5 ± 2.2	3.4 ± 2.4	<0.001
GDS score	3.4 ± 2.8	3.1 ± 2.7	3.8 ± 2.9	0.009		3.4 ± 2.6	4.8 ± 3.0	<0.001
Walking speed in m/m	68.3 ± 13.9	70.8 ± 12.4	66.2 ± 16.5	<0.001		69.0 ± 12.8	55.9 ± 15.1	<0.001
MMSE score	25.3 ± 2.9	25.2 ± 2.7	24.6 ± 3.5	0.024		25.7 ± 2.9	24.5 ± 3.5	<0.001
Word list memory task score	2.7 ± 1.9	2.6 ± 1.8	2.3 ± 1.8	0.106		3.0 ± 1.9	2.4 ± 2.1	0.003
SDST (tablet version) score	32.0 ± 8.2	33.8 ± 7.7	31.0 ± 8.6	<0.001		31.6 ± 7.9	27.3 ± 8.4	<0.001

Notes: Values are means ± SD; GDS = Geriatric Depression Scale; MMSE = Mini-Mental State Examination; TMT-B = Trail Making Test-Part B; SDST = Symbol Digit Substitution Test; m/m = meters per minute.

Table 2 shows the results of the self-reported IADL limitation survey at 15-month follow up in participants who reported no baseline IADL limitation. Rates of “no” responses were observed in 137 (14.5%), 67 (7.1%), and 119 (2.6%) participants with respect to using a bus or train, buying daily necessities, and managing deposits and savings, respectively. Rates of subsequent activity limitation differed significantly between men and women with respect to buying daily necessities (men 9.3%, women 4.8%; *p* = 0.007) and managing deposits and savings (men 17.5%, women 7.3%; *p* < 0.001). At the 15-month follow-up survey, 153 (27.4% of 485) men and 94 (20.3% of 463) women reported one or more IADL limitations and experienced subsequent IADL limitation (*p* < 0.001).

**Table 2:** Self-reported IADL limitation in the 15-month follow-up survey in participants without IADL limitation at baseline (n = 948).

IADL items	No Baseline IADL Limitation (n = 948)	Men (n = 485)	Women (n = 463)	*p*-Value
*Subsequent activity limitation (number of participants)*				
Using bus or train by myself	137 (14.5)	66 (13.6)	71 (15.3)	0.450
Going out and buying daily necessities by myself	67 (7.1)	45 (9.3)	22 (4.8)	0.007
Managing own deposits and savings at the bank	119 (12.6)	85 (17.5)	34 (7.3)	<0.001
*Number of limitations*				
None (independent)	721 (76.1)	352 (72.6)	369 (79.7)	<0.001
One	152 (16.0)	83 (17.1)	69 (14.9)	
Two	54 (5.7)	37 (7.6)	17 (3.7)	
Three or more	21 (2.2)	13 (2.7)	8 (1.7)	

Notes: Values represent number of participants (%).

Table 3 represents differences in characteristics between the independent and IADL-limitation groups. Men with subsequent IADL limitation reported lower education levels (*p* = 0.010) and demonstrated higher baseline GDS scores (*p* < 0.001), slower baseline walking speeds (*p* < 0.001), and worse baseline cognitive performance (MMSE: *p* = 0.030; word list memory: *p* < 0.001; SDST: *p* < 0.001) relative to men without subsequent IADL limitation. 

**Table 3:** Comparison of baseline characteristics between participants with and without self-reported IADL limitation in the 15-month follow-up survey (n = 948).

Variable	Men (n = 485)				Women (n = 463)		
	Independent (n = 352)	IADL Limitation (n = 133)	*p*-Value		Independent (n = 369)	IADL Limitation (n = 94)	*p*-Value
Age in years	79.0 ± 3.6	79.5 ± 4.7	0.156		78.5 ± 3.4	81.0 ± 4.6	<0.001
Education in years	11.5 ± 2.9	10.7 ± 2.9	0.010		10.2 ± 2.1	9.6 ± 2.2	0.012
Number of medications used	2.3 ± 2.3	2.7 ± 2.6	0.101		2.5 ± 2.1	3.0 ± 2.1	0.032
GDS score	2.8 ± 2.6	3.7 ± 2.9	<0.001		3.2 ± 2.5	3.9 ± 2.5	0.030
Walking speed in m/m	72.7 ± 11.6	67.6 ± 15.3	<0.001		70.6 ± 13.1	60.4 ± 13.9	<0.001
MMSE score	25.6 ± 2.7	25.0 ± 2.9	0.030		26.2 ± 2.6	24.8 ± 3.3	<0.001
Word list memory task score	2.9 ± 1.8	2.1 ± 1.7	<0.001		3.4 ± 1.9	2.2 ± 1.9	<0.001
SDST (tablet version) score	35.1 ± 7.4	31.7 ± 7.9	<0.001		33.5 ± 7.4	27.8 ± 8.2	<0.001

Notes: Values represent means ± SD or numbers of participants (%); GDS = Geriatric Depression Scale; MMSE = Mini-Mental State Examination; SDST = Symbol Digit Substitution Test; m/m = meters per minute.

There were no significant differences in age or numbers of medications used. Women with subsequent IADL limitation were older (*p* < 0.001), reported lower education levels (*p* = 0.012) and exhibited higher baseline GDS scores (*p* = 0.032), slower baseline walking speeds (*p* < 0.001), and worse baseline cognitive performance (*p* < 0.001) than women without subsequent IADL limitation during the 15-month follow-up period.

Age, sex (male), walking speed and GDS, MMSE, word list memory, and SDST scores were entered into a logistic regression model (Table 4). In men, we found that walking speed (odds ratio [OR]: 0.980, 95% CI [0.961–0.999]; *p* = 0.035) and word list memory scores (OR: 0.842, 95% CI [0.736–0.962]; *p* = 0.012) at baseline were independent predictors of subsequent IADL limitation. In women, walking speed (OR: 0.972, 95% CI [0.951–0.993]; *p* = 0.009), word list memory scores (OR: 0.833, 95% CI [0.712–0.974]; *p* = 0.022), and SDST scores (OR: 0.960, 95% CI [0.922–0.999]; *p* = 0.042) at baseline were independent predictors of subsequent IADL limitation.

**Table 4:** Baseline characteristics and cognitive function associated with subsequent self-reported IADL limitation in logistic regression.

Variable	Men (n = 485)			Women (n = 463)	
	Odds Ratio	95% CI		Odds Ratio	95% CI
Age in years	0.959	0.905–1.017		1.057	0.988–1.130
Education in years	0.964	0.889–1.044		0.991	0.877–1.119
Number of medications used	1.049	0.964–1.142		1.062	0.945–1.194
GDS score	1.060	0.979–1.148		1.018	0.923–1.124
Walking speed in m/m	0.980 *	0.961–0.999		0.972 **	0.951–0.993
MMSE score	1.010	0.929–1.099		0.980	0.890–1.079
Word list memory task score	0.842 *	0.736–0.962		0.833 *	0.712–0.974
SDST (tablet version) score	0.969	0.935–1.003		0.960 *	0.922–0.999

Notes: GDS = Geriatric Depression Scale; MMSE = Mini-Mental State Examination; SDST = Symbol Digit Substitution Test; m/m = meters per minute. * *p* < 0.05; ** *p* < 0.001.

## 4. Discussion

In this cross-sectional study, which included a large sample of adults aged 75 years and older, older adults with IADL limitations showed poorer cognitive performance including global cognition, processing speed, and memory (only in women) than those with intact IADLs. In the longitudinal analysis involving the participants without baseline IADL limitation, we confirmed that the group that had subsequently developed IADL limitation during the 15-month follow-up period exhibited poorer performance in tests assessing walking speed and cognitive function at baseline. In particular, associations between subsequent IADL limitation and baseline measures of walking speed and memory were independent of age, education, number of medications used, depression status, and global cognition in both men and women. In addition, poor processing speed was independently associated with a future decline in IADLs in older women.

Our finding that baseline cognitive function was associated with longitudinal decline in IADLs extends previous findings regarding the effects of cognitive function on the ability to function in daily life. IADL limitation may be an important predictor of mild cognitive impairment and dementia in cognitively healthy older adults [22,23]. Conversely, poor cognitive function affected the onset of IADL decline. In particular, executive and memory dysfunction may have a greater impact on future IADL limitation [8,9,24]. The results of the present study also indicate that older adults with poor performance in tests that assess memory and executive function, particularly processing speed at baseline, had a higher risk of developing subsequent IADL limitation. 

These cross-sectional and longitudinal analyses determined whether participants had experienced the onset of a decline in three IADLs, including using transportation, shopping, and managing finances. A discussion regarding the types of activity that are considered IADLs would be useful here. In older people, other activities, such as cooking, housekeeping, and handling medication, are necessary for the maintenance of independence. In addition, previous studies have divided IADLs into two categories based on differences between activities involving high or low cognitive demand [22,25]. Reppermund and colleagues suggested that the restriction of functional ability was more prominent in highly cognitively demanding activities in individuals with cognitive impairment, including that of a mild degree, relative to cognitively healthy individuals, and that relative to older women, older men seem to experience greater difficulty in the performance of IADLs that involve higher cognitive demand [25]. Furthermore, with regard to associations between cognitive function and IADL performance, processing speed was associated with the performance of IADLs involving both higher and lower cognitive demand, but memory was only associated with high-demand factors [25]. The questionnaire items used to assess IADL performance in the current study may have involved lower cognitive demand, but managing finances, in particular, seemed to require higher cognitive demand. Although it may be difficult to clarify differences in cognitive demand with respect to the IADLs assessed in the current study, our findings suggest that cognitive function, including memory and processing speed, may be an important factor in maintaining IADLs, such as using transportation, shopping, and managing finances in later life. Differences between men and women, with respect to factors associated with the onset of a decline in IADL performance, may have occurred because of differences in cognitive demand between activities.

Walking ability has been found to be a useful predictor for the onset of functional decline in older people [26]. We confirmed that walking speed was strongly associated with the ability to perform IADLs and was found to be an independent predictor for the onset of IADL decline. In the aged population, walking speed could be a valuable predictor of health status, including disability [27], falls [28], cognitive impairment [29], institutionalization [30], and survival [31]. The results of this study indicated that slower walking speed, even in independent older adults without IADL limitation, predicted subsequent activity limitation. This suggests that walking speed is worthy of being considered predictive of IADL limitation. Relative walking speed can be measured in any setting, without the need for specific skills or a particular environment. Therefore, walking speed could be considered a common variable with which to assess functional ability in older adults. In contrast, GDS score was not an independent predictor for subsequent IADL limitation in the longitudinal analyses. Some previous studies have suggested that depressive symptoms may be a predictor for an increase in disability [14,32]. The relationship between depressive symptoms and IADL limitation may be more complex [14]. This relationship is unlikely to be linear; more severe depressive symptoms (e.g., those of moderate or major depression), may have a greater impact on subsequent IADL limitation. These findings suggest that cognitive and physical health may be independent with respect to IADL in adults aged 75 years and over. The findings of this study may also assist in the better prevention system (e.g., decision support system and using exergaming) for early detection of the elderly risks like dementia and depression and improving physical fitness and life quality of elderly [33,34].

Strengths of the current study include the large sample size, the residential status and age of the participants (community-dwelling adults aged 75 or older), the duration of the study period (15 months), and the longitudinal nature of the study design. However, we acknowledge that the use of self-reported IADL function as assessed by only three items is a major limitation in this study. The assessment of subsequent IADL limitation at follow up only included three self-reported aspects of daily activity, which were using transportation (bus or train), shopping, and managing finances. In addition, the follow-up period (15 months) was shorter than that used in previous research (e.g., 2–10 years). Other IADL areas, such as using a phone, preparing meals, housekeeping, and taking medicine, should be considered as well. We excluded older individuals with a self-reported history of Parkinson’s disease, stroke, Alzheimer’s disease, or depression and included participants were who living independently in the community and did not need any personal care or support; thus, people with mild neurological disorders or mild cognitive impairment were likely to be included. A more comprehensive assessment of IADL function will be needed in future research to clarify the longitudinal relationship between decline in IADL and cognitive functioning. Furthermore, because we did not repeat measures of cognitive function or walking speed, we could not assess bidirectional associations between cognitive and physical functioning and IADL limitation. Therefore, further work is required to explore interactive associations and the effects of changes in cognitive function and walking speed on subsequent IADL limitation.

## 5. Conclusions 

The OSHPE findings demonstrated relationships between cognitive decline, impaired physical functioning, and IADL limitation. The results also indicated that walking speed, memory, and processing speed may predict subsequent IADL limitation in community-dwelling adults aged 75 years and older.
